# Changes in labial capillary density on ascent to and descent from high altitude

**DOI:** 10.12688/f1000research.7649.1

**Published:** 2016-08-30

**Authors:** Edward Gilbert-Kawai, Jonny Coppel, Phillip Hennis, Michael Grocott, Can Ince, Daniel Martin

**Affiliations:** 1 University College London Centre for Altitude Space and Extreme Environment Medicine, UCLH NIHR Biomedical Research Centre, Institute of Sport and Exercise Health, London, UK; 2 Integrative Physiology and Critical Illness Group, Clinical and Experimental Sciences, Mailpoint 810, Sir Henry Wellcome Laboratories, Faculty of Medicine, University of Southampton, University Hospital Southampton NHS Foundation Trust, Southhampton, UK; 3 Anaesthesia and Critical Care Research Unit, University Hospital Southampton NHS Foundation Trust, Southhampton, UK; 4 Department of Intensive Care, Erasmus MC University Hospital Rotterdam, Rotterdam, Netherlands; 5 University College London, Division of Surgery and Interventional Science, Royal Free Hospital, London, UK; 6 Erasmus MC-Sophia Children's Hospital, Rotterdam, Netherlands

**Keywords:** Capillaries, Microcirculation, Altitude, Microscopy, Oxygen

## Abstract

Present knowledge of how the microcirculation is altered by prolonged exposure to hypoxia at high altitude is incomplete and modification of existing analytical techniques may improve our knowledge considerably. We set out to use a novel simplified method of measuring *in vivo* capillary density during an expedition to high altitude using a CytoCam incident dark field imaging video-microscope.

The simplified method of data capture involved recording one-second images of the mucosal surface of the inner lip to reveal data about microvasculature density in ten individuals. This was done on ascent to, and descent from, high altitude. Analysis was conducted offline by two independent investigators blinded to the participant identity, testing conditions and the imaging site.  Additionally we monitored haemoglobin concentration and haematocrit data to see if we could support or refute mechanisms of altered density relating to vessel recruitment. Repeated sets of paired values were compared using Kruskall Wallis Analysis of Variance tests, whilst comparisons of values between sites was by related samples Wilcoxon Signed Rank Test. Correlation between different variables was performed using Spearman’s rank correlation coefficient, and concordance between analysing investigators using intra-class correlation coefficient.

There was a significant increase in capillary density from London on ascent to high altitude; median capillaries per field of view area increased from 22.8 to 25.3 (p=0.021). There was a further increase in vessel density during the six weeks spent at altitude (25.3 to 32.5, p=0.017). Moreover, vessel density remained high on descent to Kathmandu (31.0 capillaries per field of view area), despite a significant decrease in haemoglobin concentration and haematocrit.

Using a simplified technique, we have demonstrated an increase in capillary density on early and sustained exposure to hypobaric hypoxia at thigh altitude, and that this remains elevated on descent to normoxia. The technique is simple, reliable and reproducible.

## List of Abbreviations

EBC              Everest Base Camp

FOV              Field of view area

[Hb]              Haemoglobin concentration

Hct                Haematocrit

IDF                Incident Dark Field

KTM             Kathmandu

LON             London

SpO _2_             Peripheral oxygen saturation

## Introduction

The physiological processes involved in acclimatisation to high altitude attempt to maintain adequate oxygen delivery as the partial pressure of oxygen decreases. Traditionally, research has concentrated on global haemodynamics and the macrocirculation, variables such as cardiac output ^1^, oxygen saturations ^2^ and haemoglobin concentration [Hb] ^3^. Far fewer studies have focused on the microcirculation, which regulates blood flow to match micro-regional oxygen demand. Disruption of microvascular blood flow could explain a failure of acclimatisation in some individuals as well as the well-documented exercise limitation that occurs at altitude despite normalisation of systemic oxygen delivery ^4^. The precise role of the microcirculation in acclimatisation to hypoxia, however, remains unclear.

Teleological reasoning would suggest that increasing capillary density could provide a means to augment oxygen flux and tissue oxygenation through a reduction in the inter-capillary distance ^5^. Whilst plausible, data on this theory remains contradictory, though this may in part relate to the dissimilar tissues observed. In human skeletal muscle biopsy samples previously exposed to hypobaric hypoxia, no evidence of neovascularisation has been demonstrated ^6– 9^. Interestingly, in each instance whereby the capillary density was initially thought to increase, no change in the capillary-to-fibre ratio was observed. The perceived rise in capillary density were therefore interpreted as being secondary occurrences in response to a reduction in skeletal muscle mass. Conversely, an increase in the density of sublingual microcirculatory vessels to >25 μm was demonstrated on ascent to high altitude ^10, 11^, a response that was further amplified after prolonged exposure to hypoxia ^10^. In this instance, what remains to be determined is whether the observed changes in vessel density are due to microvascular recruitment secondary to increased blood viscosity (and thus quickly reversible), or neovascularization (which is likely to be sustained). Moreover, the question of what happens to vessel density following re-exposure to normoxia remains to be elucidated.

We therefore piloted a novel modification of a previously described technique for calculating changes in capillary density ^12, 13^ on ten individuals, to see if we could firstly support or refute previous findings on ascent to high altitude, and secondly see if the changes observed persist on descent. Additionally we monitored haemoglobin concentration and haematocrit data to see if we could support of refute mechanisms of altered density relating to vessel recruitment.

## Methods

The study was undertaken as part of the Xtreme Everest 2 research expedition (XE2) ^14^. The study design, risk management plan and protocol were approved (in accordance with the declaration of Helsinki) both by the University College London Committee and the Ethics of Non-National Health Service Human Research, and the Nepal Health Research Council (Reg no. 139/2012). Written consent was obtained from all participants. Baseline images of the labial capillaries were initially obtained from ten individuals in London (LON) (35m) in December 2012 and January 2013. Sequential images were taken after an 11 day ascent to Everest Base Camp (EBC-early) (5300m), then after 6 weeks residence at Everest Base Camp (EBC-late), and finally on descent, over 5 days, to Kathmandu (KTM) (1300m) in May 2013.

**Figure 1.**
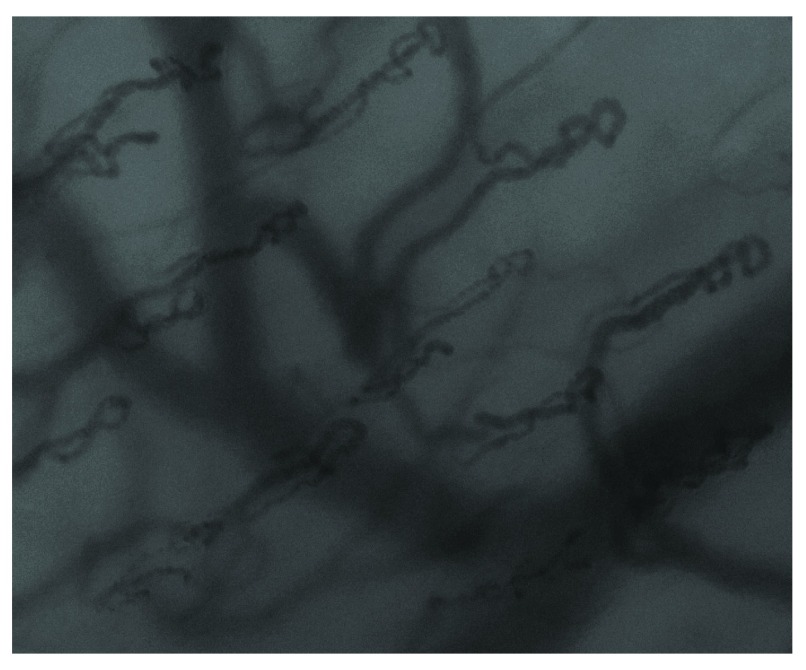
An example of a labial capillary density image.

Images were obtained using a CytoCam-IDF video microscope (Braedius, Medical BV, Netherlands). This new device is based on the principle of Incident Dark Field (IDF) imaging, which uses polarized green light (wavelength 548nm) produced from LEDs to visualize, in real time, the sublingual microvasculature. Its high resolution imaging sensor (14 Mpixel) allows for a 50% increase in optical resolution (300 lines/mm) compared to previous Sidestream Dark Field imaging devices, and it generates a far larger field of view. With the participant lying in the supine position having rested for a minimum of 10 minutes, the CytoCam-IDF device’s probe was introduced into their mouth and placed on the mucosal surface of the inner lip. Once a suitable image was visualised on the screen of the CytoCam-IDF monitor, ( Figure 1), 1 second of digital video footage was recorded. This process was conducted on all four lip quadrants (right upper lip, left upper lip, right lower lip, left lower lip), and at each quadrant four separate videos were acquired. Two trained investigators (EGK, PH) obtained all the data.

To determine capillary density, analysis was conducted offline by two independent investigators blinded to the participant identity, the testing conditions and the imaging site (EGK, JC). Using the company’s own video software (CytoCamTools V1, Braedius, Netherlands), a single still frame was projected on the computer screen and the number of capillary loops per image frame was counted manually. Partly visualised capillaries were included if the observer was assured that the vessel was a capillary due to its morphology. Subsequently, the mean capillary density was calculated from the four images obtained in each lip quadrant, and from these four results, the mean total lip density obtained. Capillary density was defined as the number of capillaries counted per field of view area (FOV), which equates to 1.79 mm ^2^. The haemoglobin concentration ([Hb]) (Hemocue AB, Hemocue, Sweden) and haematocrit (Hct) (Sigma 1–14 microcentrifuge, Sigma, Germany) were obtained from whole blood samples, and peripheral arterial oxygen saturation (SpO _2_) measured (Nonin Onyx 9500, Nonin Medical Inc, Minnesota, USA) on the same days as microcirculatory imaging was performed.

### Statistical analysis

As data were not normally distributed, they were described by median and interquartile range. Repeated sets of paired values were compared using Kruskall Wallis ANOVA, whilst comparisons of values between LON baseline and other sites was by Wilcoxon Signed Rank Test. Correlation between different variables was performed using Spearman’s rank correlation coefficient, and concordance between analysing investigators using intra-class correlation coefficient. All statistical analysis was undertaken on SPSS version 21 (SPSS Inc., Chicago, IL, USA), and a P value of <0.05 was taken to indicate statistical significance.

## Results

IDF values The number of loop capillaries seen per field of view Click here for additional data file. Copyright: © 2016 Gilbert-Kawai E et al. 2016 Data associated with the article are available under the terms of the Creative Commons Zero "No rights reserved" data waiver (CC0 1.0 Public domain dedication).

Physiological values The haemoglobin, haematocrit and peripheral oxygen saturations at each laboratory. Click here for additional data file. Copyright: © 2016 Gilbert-Kawai E et al. 2016 Data associated with the article are available under the terms of the Creative Commons Zero "No rights reserved" data waiver (CC0 1.0 Public domain dedication).

CytoCam-IDF imaging was conducted on all ten individuals on the first two occasions, however only eight individuals had data captured on descent. No problems were encountered with the device or image acquisition. Mean laboratory barometric pressure and mean temperature for each location is shown in Table 1.

**Table 1.** Mean (standard deviation) atmospheric pressure and temperature at each laboratory.

Site	Atmospheric Pressure (kPa)	Temperature (°C)	Humidity (%)
**London**	100.6 (0.2)	16.9 (1.8)	35.4 (6.5)
**Everest Base** **Camp**	53.0 (0.2)	12.9 (8.2)	37.8 (17.5)
**Kathmandu**	86.8 (0.4)	23.8 (3.4)	47.4 (15.7)

**Figure 2.**
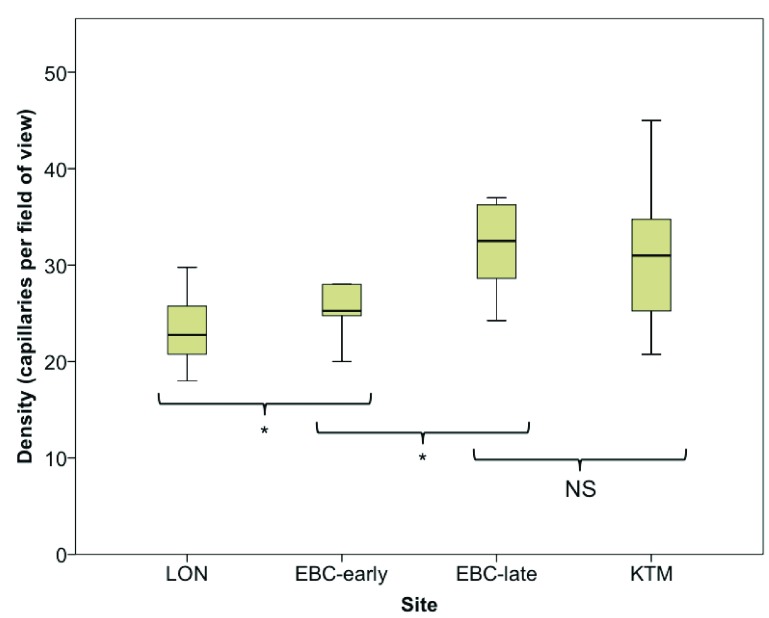
Box-whisker plots of labial capillary density on ascent from London to Everest Base Camp, after six weeks at Everest Base Camp, and on descent to Kathmandu.

Changes in labial capillary density are shown in Figure 2. Compared with LON (median 22.8 capillaries per field of view area (20.7–26.8)), capillary density was significantly increased at EBC-early 25.3 (24.5–30.6; p=0.021), EBC-late 32.5, (28.4–36.63; p=0.012), and on descent in KTM 31.0 (24.0–35.13; P=0.017). Between EBC-early and EBC-late, capillary density increased significantly (p=0.017), however there was no significant decline in density between EBC-late and KTM (p = 0.069).

Changes in [Hb], Hct and SpO _2_ at each site are shown in Table 2. There was a significant increase in [Hb] between LON and EBC-early (p=0.007), and EBC-early and EBC-late (p=0.011), and a decrease between EBC-late and KTM (p=0.008). There was also a significant increase in Hct between LON and EBC-early (p=0.007), and EBC-late and KTM (p=0.012), but no significant change between EBC-early and EBC-late (p=0.191). Between the sites on ascent, the increase in vessel density demonstrated an inverse relationship with the SpO _2_, however at each altitude there was no correlation between vessel density and [Hb], Hct or SpO _2_.

**Table 2.** Median (interquartile range) haemoglobin concentration, haematocrit and peripheral oxygen saturations at each measurement point.

Site	Hemoglobin concentration (g/dl)	Hematocrit (%)	Oxygen saturation (%)
**London**	14.5 (13.5–15.3)	44.0 (41.5–47.5)	99 (98–100)
**Everest Base** **Camp - early**	16.4 (15.7–17.2)	52.0 (51.5–56.3)	81 (79–86)
**Everest Base** **Camp - late**	18.3 (17.1–19.7)	56.0 (51.0–61.5)	88 (85–91)
**Kathmandu**	16.0 (14.0–17.0)	50.0 (46.5–55.0)	99 (99–100)

To assess whether an image capture time of 1 second was indicative of that captured over longer periods of time, we obtained 30 seconds of footage from four individuals at two different locations. From this we randomly selected one frame per five seconds of footage, and counted the number of capillaries per field of view area. The values of these may be seen in Table 3, as too can the mean and standard deviations for each set of frames, the latter of which demonstrates a highest value of only 0.52 capillaries per field of view area.

**Table 3.** Number of capillaries counted per field of view area from one randomly selected frame per five seconds of footage, with mean and standard deviation demonstrated.

Code	Site	Frame 1 (0–5 s)	Frame 2 (5–10 s)	Frame 3 (10–15 s)	Frame 4 (15–20 s)	Frame 5 (20–25 s)	Frame 6 (25–30 s)	Mean	SD
1	LON	30	30	30	30	30	30	30.0	0.00
2	LON	24	25	24	24	24	25	24.3	0.52
3	KTM	27	27	27	27	26	27	26.8	0.41
4	KTM	22	23	23	23	23	22	22.7	0.52

s = seconds; SD = standard deviation

## Discussion

This study demonstrates for the first time, persistence of *in vivo* sublingual microvascular density increase on re-exposure to normoxia after a prolonged period of hypobaric hypoxia at high altitude. We utilised an infrequently used imaging and analysis technique that we had purposefully adapted to suit our needs, and found our data aligned with previously published work on blood vessel density at altitude ^10^.

Using the data obtained from corresponding blood samples, it is possible to speculate on the adaptive processes occurring at each measurement point. As previously described, we observed a significant rise in [Hb] (14.5 g/dl to 16.4g/dl; p=0.007) and Hct (44% to 52%; p=007)) on ascent to altitude. Whilst this polycythaemia increases arterial oxygen content, blood viscosity also rises, altering its rheology. Under normal physiological conditions, a considerable proportion of the microcirculation is thought to be ‘unrecruited’, acting as a reservoir for times of increased metabolic needs ^15^. As Hct rises, these reserve vessels are recruited, and microvascular density increases, along with functional capillary density ^15– 18^. Thus a secondary benefit to increased Hct is achieved; a reduction in the diffusion distance from capillaries to mitochondria. Importantly however, it should be noted that in normal capillary Hct is generally 50% less than systemic Hct owing to the streamlined blood flow in narrow capillaries ^19^. The effect of hypoxia on this association is unknown. Whether or not neovascularisation had occurred on arrival at high altitude is difficult to say, although due to the short time between measurement points the chances of this being the case are low ^20^.

After 6 weeks spent at altitude, a further, and far greater, increase in vessel density was apparent; EBC-early 25.3 capillaries per field of view area, EBC-late 32.5 (p=0.017). Over the same time period, [Hb] had significantly risen, whilst Hct had not. As Hct is a more reliable indicator of viscosity between the two variables ^21^, it seems unlikely that further recruitment of the microvasculature had occurred, yet it is plausible that neovascularisation had. Increased levels of vascular endothelial growth factor (VEGF) have been detected in subjects ascending to high altitude ^22^; its role in angiogenesis perhaps explaining the observed rise in microvascular density ^10^. Such adaptations lead to improved tissue oxygenation by a reduction of the inter-capillary distance, whilst maintaining a sufficiently low, and thus fluid Hct to permit flow of red blood cells in the microvasculature.

On descent to a lower altitude (KTM) there was no significant fall in microvascular density when compared to EBC-late (p = 0.069), however, a much greater number of vessels (36% increase) was evident when compared with baseline testing in LON. Whilst vessel density was thus unaltered on descent, over the same time point [Hb] and Hct values significantly declined (p=0.012). When compared to the original LON values, Hct on descent to KTM was significantly higher (44.0% and 50.0% respectively (p=0.011)), however, [Hb] was not (14.5 and 16.0g/dl (p=0.052)). Teasing apart the relative contributions of vessel recruitment and neovascularisation to the observed changes in sublingual microcirculatory density is challenging. Whilst the failure of vessel density to return to baseline after descent suggests some neovascularisation, neither [Hb] nor Hct had normalised at the time of the final readings so a raised blood viscosity could perhaps be maintaining a heightened level of capillary recruitment. A combination of the two processes would make sense as continually increasing [Hb] to improve oxygen delivery would eventually be counter productive. Indeed in Tibetans, who have been exposed to environmental hypoxia for many generations, there is a clear reduction in [Hb] compared to populations who have been exposed to these conditions for less time ^23– 26^. This suggests Tibetans utilize alternative long-term strategies for chronic adaptation to hypobaric hypoxia, ones that do not rely on maintaining a high [Hb]. It is plausible that one such means would be to increase their capillary density.

### Technique and analysis

The use of the described methodology was also novel. A similar technique has been used twice previously; once in the assessment of coronary artery disease in diabetes ^13^ and the other in a study investigating hypertension and rarefaction during treatment with Telatinib ^12^. In these instances, data capture involved recording sublingual images for 1 minute ^13^ or 30 seconds ^12^ per quadrant, however we altered this time period by using an extremely short capture phase for data acquisition (< 1 second). Crucially, this allowed us to readily obtain snap shot images to reveal data about microvasculature density, whilst avoiding concerns surrounding probe and patient movement, in addition to issues relating to pressure artefact. Analysis was rapid, simple and reproducible; in this study it had an observer mean intra-class correlation coefficient of 0.91 (95% CI 0.84 – 0.96). Previously no difference in capillary density was observed in ten individuals between lip quadrants, and the reproducibility of the technique to determine capillary density was moderate to high with a coefficient of variation of 4.6% ^12^. Of note, the technique does not allow assessment of microvascular flow, nor does it yield information on heterogeneity of microvascular blood flow, however, ** we propose it to be a robust method for the assessment of labial vessel density that could be conducted after only a short user training period.

### Study limitations

The small number of participants used in this study could be considered a study limitation. As we were both employing a newly-adapted data acquisition technique, and using a novel device at altitude, no power calculation was performed. This therefore increases the risk of a type 2 error. Other limiting factors include the environmental considerations associated with high altitude research in a remote field environment. These include fluctuations in laboratory temperature, humidity ( Table 1) and participant hydration status, all factors that may alter microvascular blood flow and density. Attempts were made to limit these potential confounding factors by performing CytoCam-IDF imaging at the same time of day in heated purpose-built laboratories, and encouraging participants to maintain a good state of hydration. Previous studies at altitude have also raised concerns over the development of tissue oedema ^10, 11^ that can occur on ascent to altitude ^27^. Whilst this could potentially reduce image quality and lead to false measurements of flow and density, our IDF camera provided us with a depth of focus reading, thus allowing us to confirm that we were recording at the same depth under the tongue on each time point. Finally, we have discussed alterations in capillary or vessel density. It is important however to clarify this nomenclature. IDF imaging cannot image blood vessels directly but rather uses the fact that polarized green light is optimally absorbed by red blood cells within the microvasculature regardless of oxygenation status. Absorption of light by haemoglobin, but not by surrounding tissues, therefore creates a distinct contrast of dark and light colour respectively, and red blood cells moving through the mucosal microcirculation thus appear as dark globules moving along the axis of flow. All vessels visualized are therefore only seen if they contain erythrocytes. The variables measured by IDF imaging (and its precursor SDF imaging) include a measure of total vessel density (TVD) and perfused vessel density (PVD) ^28^. A distinction is made between the two depending on the speed of red blood cell flow within the observed vessels. TVD includes vessels which contain erythrocytes flowing at any velocity (or even at standstill), whilst PVD only includes vessels with continuously moving erythrocytes. As we cannot measure erythrocyte velocity with this simplified method, our observations therefore describe the TVD.

## Conclusions

This study demonstrated an increase in sublingual microvascular vessel density on early and sustained exposure to hypobaric hypoxia; and, for the first time, that no significant change in vessel density occurred on immediate descent. The technique used to capture the images provided a rapid and reliable means for assessing changes in vessel density, and could be applied in future studies of microcirculatory vessel density. Further research in this area may allow a more complete comprehension of the multidimensional response to sustained hypoxia that occurs during pathophysiological situations.

## Data availability

The data referenced by this article are under copyright with the following copyright statement: Copyright: © 2016 Gilbert-Kawai E et al.

Data associated with the article are available under the terms of the Creative Commons Zero "No rights reserved" data waiver (CC0 1.0 Public domain dedication). 

*F1000Research*: Dataset 1. IDF values, 10.5256/f1000research.7649.d134021 ^29^

*F1000Research*: Dataset 2. Physiological values, 10.5256/f1000research.7649.d134022 ^30^
