# Supplementary material for: Changes in labial capillary density on ascent to and descent from high altitude
Source: F1000Res. 2016 Aug 30;5:2107. [Version 1] doi: 10.12688/f1000research.7649.1 (PMC5043444; doi:10.12688/f1000research.7649.1)
Supplement: Peer review history

# Peer review: Referee response for version 1

**DOI:** 10.5256/f1000research.8238.r15955

**Published:** 2016-09-28

**Authors:** Matthias Hilly

**Affiliations:** 1 Medical Intensive Care Unit, University Hospital of Zurich, Zurich, Switzerland

The authors report on the use of a relatively novel method to determine capillary density during acute and chronic exposure to hypobaric hypoxia. Previously reported results of an increase in capillary density during acclimatization are confirmed, but it is for the first time demonstrated that upon immediate descent some changes persist.

 Overall, this is a well-conducted study that adds to the current knowledge base of microcirculatory changes during adaptation to hypobaric hypoxia, and provides an innovative approach to vessel density measurement.

 Specific comments:

**Introduction**

 In the introduction the absence of neovascularization in muscle samples during adaptation to high altitude is discussed. It is however also known that mitochondrial density increases during this process (PMID 26339730). The authors may want to include this in the discussion about capillary-to-fibre ratio, the mitochondria arguably representing the circulation's endpoint.

**Methods**

 The KTM time point seems difficult to me for use as a “post-hypoxia” measurement, since it is not comparable to the LON baseline. This is not a severe limitation since “immediate descent” was one of the study endpoints rather that a comparison to the initial (LON) baseline. I still suggest mentioning this in the limitations.

 Microcirculation capture time is very short (1 sec). I am not sure if the analysis in four subjects given in table 3 is sufficient support for the use of much shorter capture intervals than recommended by current consensus guidelines. This could be discussed in more detail in the limitations.

 Hb and Hematocrit values, as well as SpO _2_ were determined “on the same day as microcirculatory imaging was performed”. Given the influence of hydration status changes throughout a day, especially in a mountain setting, either specify the time frame more clearly or mention this time relation in the limitations.

**Results**

 Figure 2: It is unclear to me what the results of the Kruskal-Wallis ANOVA described in the methods section were for vessel density measurements, consider mentioning these in the results section. Further, are the P values given in the text references to Wilcoxon Signed Rank tests as mentioned in the methods section? Consider describing these tests as post-hoc analysis for the non-parametric ANOVA. And if so, was a correction for multiple testing applied (Bonferroni, or one of the newer algorithms)? Please describe this more clearly.

 It is mentioned that at each altitude there was no correlation between vessel density and Hb, Hct or SpO _2_. In the methods section it is stated that Spearman's rank correlation coefficient was calculated. Consider adding correlation coefficients and P values to the results section to provide the readers some insight into the data.

 In addition to Hb, Hct and SpO _2_ given in Table 2, it would be of interest to know more about the subjects' physiologic parameters, such as blood pressure, cardiac output and even RV/RA – including a discussion about the changes in macrocirculation in relation to the changes in microcirculation.

**Discussion**

 Given borderline significance (p=0.069) for a decrease in vessel density from EBC-late to KTM and the lack of a power calculation, as well as the lack of real comparability to the initial (LON) baseline it is somewhat difficult to argue that there really was no change between EBC-late and KTM. This is discussed in the discussion to some extent, but it should be stated more clearly that a verification of these results is needed in the future.

 The method for vessel density estimation used in this study differs from the effective calculation of total vessel density (TVD), being the total length of small vessels divided by the surface area covered in the recording, that is recommended by current consensus guidelines. Please discuss the relationship between vessel density as measured in this study with vessel density as assessed by TVD, and potential confounders.

I have read this submission. I believe that I have an appropriate level of expertise to confirm that it is of an acceptable scientific standard.

# Peer review: Referee response for version 1

**DOI:** 10.5256/f1000research.8238.r16269

**Published:** 2016-09-14

**Authors:** Sam Hutchings

**Affiliations:** 1 Department of Critical Care, Kings College London University, London, UK

Edward Gilbert-Kawai and colleagues involved in the Xtreme Everest 2 study report further work on the effects of altitude and hypoxaemia on the microcirculation. They describe a technique for rapidly measuring microcirculatory capillary density, using a method of counting the number of capillary loops in the buccal mucosa of healthy volunteers at sea level, progressive altitude acclimatisation and descent. They also report systemic haemoglobin and haematocrit values in an attempt to elucidate whether changes in microcirculatory parameters are paralleled by changes in blood viscosity. Their results show, in line with previous work, that microcirculatory vessel density increases after prolonged exposure to hypoxic conditions. However, they also show that there is some persistence of this effect on partial descent. The authors pose a question as to whether this increased vessel density is due to neo-vascularisation or recruitment of existing capillaries secondary to an increase in haematocrit. 

 This is generally a scientifically sound paper and a well written manuscript. I have several comments for the authors:

 Major Comments

- It strikes me that the authors are trying to achieve two things in this paper. Firstly, to confirm that there is microcirculatory adaptation to altitude and hypoxaemia and to provide more information regarding the mechanisms surrounding this and secondly to describe a method of rapidly assessing vessel density. Both subjects are not quite covered in enough detail to do either justice.
- With regard to the assessment method, this has been described previously in both buccal mucosa but also in other areas such as the nail bed. It has the advantage of being quick to perform but only provides information on one variable – number of capillary loops. The authors state and assume that this measure equates to Total Vessel Density (TVD) but it would be very useful to confirm this by actually measuring capillary TVD using sublingual IDF video capture and analysis. More importantly, the described method provides no measure of microcirculatory flow. This is crucial when discussing the impact of capillary haematocrit and viscosity on microcirculatory performance. Personally I think the paper would have been improved had the investigators used sublingual images and analyzed these to produce data on a fuller range of flow and density parameters.
- Although the authors give two possible mechanisms for the observed increase in capillary density, I would be interested to know if these changes could possibly be accounted for by an increase in systemic haemodynamic parameters, such as cardiac output. Unless there is a loss of haemodynamic coherence one would expect that micro would follow macro. Measurement of these values using a non invasive technique such as trans thoracic echocardiography would have been interesting and added value to the paper.
- The authors discuss that systemic haematocrit and haemoglobin do not necessarily equate with capillary haemoglobin. This is a crucial point and one often observes that “shocked” capillaries have a visibly lower number of erythrocytes even in the presence of a reasonably preserved systemic Hb level, especially post resuscitation. Being able to quantify this difference would allow us to answer crucial questions but is not possible with current analysis methods using this technology.
- I would be very interested to know how long this microcirculatory adaptation lasts. Did the authors consider repeating the measures after return to sea level and after a more prolonged period – e.g. one month?

 

 Minor Comments

- Southampton is spelt incorrectly in several places within the list of authors
- Citing a validation paper of the IDF camera (e.g. Hutchings S, Watts S, Kirkman E. The Cytocam video microscope. A new method for visualising the microcirculation using Incident Dark Field technology. Clin Hemorheol Microcirc. IOS Press; 2015 Oct 16;62(3):261–71) would be useful as the authors have stated that it is a new device.

I have read this submission. I believe that I have an appropriate level of expertise to confirm that it is of an acceptable scientific standard.
